# A spectrum of methods for a spectrum of risk: Generating evidence to understand and reduce urban risk in sub‐Saharan Africa

**DOI:** 10.1111/area.12510

**Published:** 2018-12-09

**Authors:** David Dodman, Ibidun Adelekan, Donald Brown, Hayley Leck, Mtafu Manda, Blessing Mberu, Mark Pelling, Maria Rusca, David Satterthwaite, Faith Taylor

**Affiliations:** ^1^ International Institute for Environment and Development London UK; ^2^ University of Ibadan Ibadan Nigeria; ^3^ University College London London UK; ^4^ King's College London London UK; ^5^ Mzuzu University Mzuzu Malawi; ^6^ African Population and Health Research Centre Nairobi Kenya; ^7^ Department of Earth Sciences Uppsala University Uppsala Sweden; ^8^ Centre of Natural Hazards and Disaster Science Uppsala Sweden; ^9^ Department of Geography University of Portsmouth Portsmouth UK

**Keywords:** Africa, governance, mixed methodologies, resilience, risk, urban areas

## Abstract

Many African towns and cities face a range of hazards, which can best be described as representing a “spectrum of risk” of events that can cause death, illness or injury, and impoverishment. Yet despite the growing numbers of people living in African urban centres, the extent and relative severity of these different risks is poorly understood. This paper provides a rationale for using a spectrum of methods to address this spectrum of risk, and demonstrates the utility of mixed‐methods approaches in planning for resilience. It describes activities undertaken in a wide‐ranging multi‐country programme of research, which use multiple approaches to gather empirical data on risk, in order to build a stronger evidence base and provide a more solid base for planning and investment. It concludes that methods need to be chosen in regard to social, political economic, biophysical and hydrogeological context, while also recognising the different levels of complexity and institutional capacity in different urban centres. The paper concludes that as well as the importance of taking individual contexts into account, there are underlying methodological principles – based on multidisciplinary expertise and multi‐faceted and collaborative research endeavours – that can inform a range of related approaches to understanding urban risk in sub‐Saharan Africa and break the cycle of risk accumulation.

## Abstract 2

Many African towns and cities face a range of hazards, which can best be described as representing a “spectrum of risk” of events that can cause death, illness or injury, and impoverishment. This paper provides a rationale for using a spectrum of methods to address this spectrum of risk, and demonstrates the utility of mixed‐methods approaches in planning for resilience.

## 1 INTRODUCTION

Many sub‐Saharan African towns and cities are risky places (Adelekan et al., 2015; Bull‐Kamanga et al., 2003). Dense concentrations of people and economic activities, high levels of water and air pollution, and inadequate provision of basic services and risk‐reducing infrastructure result in high levels of physical injury, serious illness, reduced productivity and loss of life (Hardoy et al., 2001; McGranahan et al., 2001). Most such risk – whether from disasters (Douglas et al., 2008) or from disease (Ezeh et al., 2017) – disproportionately impacts low‐income groups. Towns and cities in the region are growing rapidly (although the overall rate of growth of nations’ urban populations has been slowing in recent years), meaning that an ever‐larger number of people live in urban contexts. Smaller urban centres with fewer than 1 million inhabitants are among the fastest growing, although research has overwhelmingly focused on the largest cities (Satterthwaite, 2016).

This expansion in urban populations is not matched by an equivalent expansion in resources, capacity and appropriately located, properly serviced and affordable land and housing for low‐income groups. This means that many urban residents live (and are likely to continue living) in what can be termed slums – indeed, UN Habitat (2013) figures suggest that this is the case for 61.7% of the urban population in Africa. In addition, this urban growth is taking place alongside climate change and other pressing global challenges, such as the threat of new and emerging diseases. The IPCC Fifth Assessment Report recognises the “rapid growth of highly vulnerable urban communities living in informal settlements, many of which are on land at high risk from extreme weather” (Revi et al., 2014, p. 538). Taken together, this means that low‐income urban residents are forced to contend with multiple, sometimes overlapping, risk environments.

This paper describes and reflects on methodological approaches used in a large multi‐disciplinary, multi‐country programme of research and capacity building – Urban Africa: Risk, Knowledge (Urban ARK)^1^ – as the basis for understanding risk in urban centres in sub‐Saharan Africa. Urban ARK works in nine cities across eight countries to address gaps in data, understandings and capacity in order to break cycles of risk accumulation through partnerships between researchers, practitioners and city‐ and community‐level activists (Leck et al., 2018). In each location, Urban ARK aimed to identify the spectrum of contemporary risks, who is most at risk and the strategies that are required to address these risks (Satterthwaite et al., 2018). It covers a range of cities in different contexts – from the small but rapidly growing urban centre of Karonga in northern Malawi to the large and economically significant cities of Nairobi and Dakar. Throughout there was a focus on learning from local authorities and civil society groups while simultaneously building their capacities to manage risk more effectively, as well as on strengthening the skills of early career researchers in institutions across sub‐Saharan Africa.

Building on the experiences from this project, the paper argues for the importance of taking a “spectrum of risk” approach to understanding the events and processes that affect health, disrupt lives and livelihoods, prevent some people from escaping from poverty, and cause other “precarious” individuals and households to slip back into poverty. It concludes that as well as the importance of taking individual contexts into account, there is a need to integrate methods to support joined‐up assessments that enable complex risk to be analysed and addressed. These principles are relevant for scholars and policy makers who are faced with the methodological and practical challenges of capturing a spectrum of risk characterised by different spatial‐temporal distributions and degrees of intensity.

## 2 THE SPECTRUM OF URBAN RISK

Residents of urban centres – and particularly residents of low‐income neighbourhoods – face a range of risks. While for many years studies of disaster‐risk focused primarily on large single‐hazard disasters that caused substantial damage to property or loss of life, there has been growing recognition of extensive risk associated with the “dispersed and recurrent occurrence of small and medium scale impacts” (Lavell & Maskrey, 2014, p. 271) that cumulatively erode coping capacities and livelihood strategies. Direct engagement with residents of low‐income urban communities frequently highlights the many factors that can cause loss or damage to property, injury, ill‐health or even death, and the many other underlying drivers that can create susceptibility to harm in the multi‐hazard environments of everyday life. It also emphasises the ways in which both community and individual characteristics (including age and gender) contribute to differential patterns of risk.

The concept of the spectrum of risk has particular value in addressing these challenges in a more integrated manner. This can be understood as “all the potential and likely causes of events resulting in premature death, illness or injury, and impoverishment” (Satterthwaite & Bartlett, 2017, p. 3). Several dimensions of the spectrum of risk are particularly relevant in urban settings in low‐ and middle‐income countries. The full spectrum of urban risk also needs to consider risk associated with the lack of provision of adequate infrastructure (such as drainage), with dense poor‐quality housing (as is frequently found in informal settlements) and with the governance arrangements in urban centres (particularly where these do not adequately engage with the priorities of low‐income groups).

At one end of the spectrum of risk are the everyday health risks faced by residents of low‐income and informal settlements. Most informal settlements are particularly unhealthy places with especially high risks of infection and injury, particularly for children, with diseases such as typhoid, hookworm and cholera being particularly prevalent (Ezeh et al., 2017). At the other end are the large and highly visible urban disasters: epitomised by Hurricane Katrina in New Orleans (2005) and the Port‐au‐Prince earthquake (2010). Falling somewhere in‐between are the floods (Douglas et al., 2008), fires (Twigg et al., 2017) and temperature extremes (Klinenberg, 2001) that particularly affect low‐income and informal settlements, yet most of these are ignored outside of (or even in) local media outlets. However, a focus on everyday risk is particularly critical in sub‐Saharan Africa as a means of contributing to social justice goals in cities that are often profoundly unequal (Ziervogel et al., 2017).

What all sections of this spectrum have in common is the role of economic, social and infrastructural systems in creating – or failing to reduce – risk. The focus of Urban ARK was explicitly on risks related to land and climate, but it is recognised that a range of other systemic risks shape and are shaped by these, including terrorism, violence, crime and disruption to infrastructure and technological systems (Mitra et al., 2017). Despite these many obvious and intersecting hazards, and the lack of financial and technical capacity to minimise the harm that they cause, the legacy of a hazard‐centric paradigm to data collection continues to limit approaches to quantify and explain risk. In contrast, a focus on the spectrum of risk enables more critically engaged policy‐ and practice‐relevant research that tackles major global challenges.

## 3 FILLING THE GAP: APPROACHES TO ADDRESSING THE METHODOLOGICAL DEFICIT

Generating comprehensive, detailed and socially and spatially dis‐aggregable information on the full spectrum of risk requires reports of all events that result in premature death, serious illness or injury, or loss of or damage to assets (including housing) and livelihoods. Yet many of the records providing this type of information that are normally available in wealthier cities are absent or partial in many sub‐Saharan African cities (Osuteye et al., 2016). Through the Urban ARK programme, a range of approaches have been adopted to strengthen understanding of urban risk and generate information that is of practical relevance to local, national and global policy makers to address this. A major focus has been on engagement with city stakeholders and supporting demand‐driven approaches to evidence generation. This is explained in further detail throughout the paper and has been done through in‐depth research and policy engagement, initially in six cities – Dakar (Senegal), Niamey (Niger), Ibadan (Nigeria), Nairobi (Kenya), Mombasa (Kenya) and Karonga (Malawi) – but subsequently in additional urban centres including Freetown (Sierra Leone), Dar es Salaam (Tanzania) and Addis Ababa (Ethiopia). The approach taken throughout this multi‐country, multi‐disciplinary programme of work was deliberately open and iterative rather than narrowly defined and restrictive. While this created some limitations in terms of direct comparability, it did ensure that the most appropriate approaches – using available data, presence of local expertise and identified need from policy actors – were used in each location. The sections below provide an approximate – but useful – grouping of the types of methods that were used, describe their application and assess the contribution they make to a fuller understanding of urban risk. Taken together, these methods cover a broad range of the spectrum of hazards, vulnerabilities and risks (everyday, small and large) and demonstrate a move towards more granular and detailed data that can be triangulated and contextualised in relation to other sources of information.^2^ The following sections engage specifically with three of the areas in which Urban ARK researchers (in association with local civil society and local authorities) focused attention for the generation and analysis of data: documentary and institutional analysis, community data and participatory approaches, and observational tools and remotely sourced data.

## 4 DOCUMENTARY AND INSTITUTIONAL ANALYSIS

Despite the widespread deficiencies in records, documentary analysis can be a useful source of information on the spectrum of risk in African cities. The Demographic and Health Survey (http://dhsprogram.com) is a large‐sample survey that is undertaken on a regular basis (often every five years) to provide data for a wide range of indicators in the areas of population, health and nutrition. DHS data have been used in Urban ARK countries, including Malawi, to attempt to uncover differences between smaller and larger urban centres, from which initial observations suggest that smaller urban centres have lower provision for basic health promoting services (such as water, sanitation and electricity) than larger urban centres. However, as the DHS is sample‐based, it often does not adequately cover informal or low‐income settlements (APHRC, 2014).

One of the most comprehensive datasets available for understanding extensive risk is that generated by DesInventar (www.desinventar.net), an online database that builds a body of evidence on disasters, based on pre‐existing official data, academic records, newspaper sources and institutional reports. It initially began in Latin America in the 1990s, but has subsequently been applied in more than 90 countries around the world. An analysis of DesInventar data from Kampala, Nairobi, Niamey, Dakar and Freetown (Osuteye et al., 2016) suggested that the major losses in urban disasters were from flooding, epidemics, fires and accidents, and to a smaller extent structural collapse, industrial disasters, drowning and storms. In terms of houses destroyed or damaged, flooding is by far the most prevalent cause, although fires and storms are also causing losses. The approach was also applied directly in Ibadan to build a database of impacts from everyday hazards and disasters in the city with the aim of both improving and updating the risk data. However, DesInventar does not usually report on “everyday” human losses related to endemic conditions caused by environmental factors, such as deaths from diarrhoeal disease (unless it is classified as an outbreak, such as cholera), malaria or deaths resulting from a lack of medical attention and morbidity (Osuteye et al., 2016).

Many other sources of data can help to complement the sources above and give a fuller picture of the drivers of risk – for instance, hospital records, records of traffic accidents and records of fires. These are often difficult to obtain by outside researchers, but in the absence of comprehensive recording systems can be an effective guide to locally significant risks. For example, research in Karonga Town included archival research of inpatient records at Karonga District Hospital for a one‐year period to assess the relative importance of different environmental problems – ranging from poor water and sanitation, to seasonal floods and drought, to large‐scale disaster events – for health.

Policy and institutional analysis can highlight areas of risk that are not covered in legislation – or that are covered but are not being addressed. Haregu et al. (2017) used this approach to analyse solid waste management policies and their implementation in Kenya, and to assess how these can contribute to risk and risk reduction, particularly for low‐income urban residents – poor solid waste management is linked to gastro‐enteritis, typhoid fever, helminths, and viral and bacterial infections. This analysis demonstrated how policy changes can help to promote good working practices, but also cautioned that weak institutional structures and low levels of enforcement limit the effectiveness of these policies in reducing risk. In Lilongwe, an institutional analysis of the water sector helped build understanding of how authority relations at different scales and between different actors (individuals, public and private organisations, community based organisations etc.) co‐determine uneven access to basic services – which in turn plays a key role in shaping the spectrum of risk. In high‐density low‐income areas served through water kiosks, traditional elites such as chiefs, religious leaders and other prominent community members mobilise their authority to gain a prominent role in the Water Users Associations (WUAs), set up to manage water sales and revenue collection at the water kiosk. This institutional set‐up works to exacerbate uneven distribution of networked water supply, with the urban poor suffering the most from water stress (Alda‐Vidal et al., 2018) and risks of water contamination (Sarpong Boakye‐Ansah et al., 2016).

## 5 COMMUNITY DATA: SURVEYS AND PARTICIPATORY APPROACHES

Various forms of empirical data collection can be used to build understanding of risk in low‐income urban neighbourhoods. These approaches can vary from highly quantified social surveys to more ethnographic and participatory approaches, including some drawing on a long tradition of participatory appraisal originally developed in rural areas (Pretty et al., 1995).

Household surveys to assess risk can draw on and develop existing methodological approaches. Boubacar et al. (2017) modified the Household Economy Approach (HEA) – a tool routinely used to monitor household‐level vulnerability to food‐security shocks in rural sub‐Saharan Africa – to assess the absorptive capacity of residents in flood‐prone neighbourhoods in Niamey, Niger. This approach enabled the construction of different “resilient classes” based on security, education, economic assets, food security, health, shelter and social support. In Karonga a household survey focused more on the perception of risks (Manda & Wanda, 2017) and showed that the most commonly identified risks were those related to flooding from the river/lake, droughts/food insecurity and earthquakes. The same survey also helped to demonstrate the widespread nature of illness related to environmental conditions, with cholera and malaria being particularly frequently identified.

Surveys of this sort can draw out the risk implications of existing urban issues, such as hygiene and conflict. Rusca et al. (2017) used a household survey, semi‐structured interviews and focus groups to understand preferences and motivations around hygiene in Lilongwe. The use of these methods helped to build an understanding of both the direct benefits of hygiene practices and the co‐benefits that they can produce for reducing health risk in low‐income urban areas. Another sensitive issue that can be investigated in this way that contributes to risk and resilience is the relationship between social cohesion and risk: Mitra et al. (2017) used individual interviews and focus groups to show how slum upgrading can reduce conflict, crime, insecurity and flood risks in Kibera, Nairobi – a wide range of inter‐related risks affecting low‐income residents. The use of these types of surveys can thus help to understand local perceptions of risk, hidden risks that may not be as widely understood and the potential for building resilience. The targeted nature of these surveys means that they cover the risks facing groups – particularly those living in undocumented or informal settlements – that are often undocumented or ignored.

These local and participatory methodologies aim to combine a degree of stakeholder ownership with data collection to support local reflection and action planning (Pelling, 2007), and have proliferated recently through the work of community‐based and non‐governmental organisations working with local at‐risk populations. The Urban ARK project has not only used these approaches to record perceptions of risk, but has used them to bring a stronger understanding of the local manifestations of risk to policy attention in all the cities. While these approaches often provide nuanced insights into complex risk contexts, taken on their own they can be idiosyncratic in design and miss an opportunity for synthetic analysis. As Castán Broto argues:

The major challenge is to move away from the instrumental use of participatory methods for governance, toward deliberative approaches that recognise both the multiple capacities of urban actors and their right to participate in the making of sustainable urban futures. (2017, p. 6)

In response to this challenge and linked to the centrality of stakeholder ownership, the programme prioritised community‐led and ‐owned data collection. In Karonga, community representatives led a process of identifying and prioritising key risks, which were then used to develop an “action plan” that formed the basis for engagement with local government and other actors. This work was further developed through “ReMapRisk” – a community‐led methodology developed by Urban ARK and applied in two city contexts (Karonga and Freetown) as a means of mapping and analysing often under‐recorded everyday risks, such as water‐ and sanitation‐related diseases, and small‐scale episodic disasters, such as fires and localised floods (Allen et al., 2018). The tool allows local communities to document and monitor how risk‐accumulation cycles or “urban risk traps” materialise over time and space, feeding spatial and temporal details into an interactive online database.

These combined methodologies have helped to inform community‐led projects that engage effectively with the full spectrum of risk, such as water, sanitation and hygiene initiatives in both Sierra Leone and Karonga. Furthermore, Urban ARK researchers are working closely with Kenya's Slum‐Dweller Federation (in Kiswahili, *Muungano wa Wanavijiji*) in Mukuru informal settlement in Nairobi to integrate disaster risk‐reduction considerations into the Mukuru Special Planning Area (SPA), underpinned by community‐led data collection and profiling (Pelling et al., 2017). Such integrative approaches, which place the diverse capacities and knowledge bases of local urban actors at the centre, open up potential for transformative change and addressing underlying drivers of risk. These collaborative and community‐led approaches can help to identify policy‐ and practice‐relevant data, as well as support capacity and sustained action that exists beyond specific research projects or interventions and to create platforms for advocacy and cross‐scalar engagement between city and local actors.

In addition to local knowledge, the global citizen science community can also contribute to generation of data useful for understanding exposure to natural hazards. An Urban ARK “mapathon,” organised in London in March 2016 by King's College London Humanitarian Mappers (https://kcl-humanitarian-mappers.com/), aimed to enrich the globally crowdsourced OpenStreetMap (www.openstreetmap.org) spatial dataset of roads and buildings for the town of Karonga. Before the mapathon, 75 buildings and 243 km of roads were mapped. After the mapathon (and the following weeks where the task was available online for the global community to contribute to) 29,030 buildings and 1,347 km of road were mapped, with the data being freely available for download. As the generated map is based on tracing buildings and roads from recent aerial imagery, the resulting map presents a more neutral view of the spatial distribution of both formal and informal houses and roads across the area from which exposure to hazards such as floods and earthquakes can be better understood through overlaying hazard maps with the building and road infrastructure maps.

## 6 OBSERVATIONAL TOOLS AND REMOTELY SOURCED DATA

Technological advances have made it increasingly easy to record different types of data using relatively low‐cost and portable equipment. Much of this is related to capturing spatial locations of particular phenomena, and to more easily record accurate data on air and water pollution. While the mechanisms linking exposure to solid waste and health risk are clear (Ziraba et al., 2016), the extent to which this results in physical harm in cities in sub‐Saharan Africa is less well quantified. The use of epidemiological and demographic quantitative and qualitative studies in Mombasa and Nairobi (Kenya) and Dakar (Senegal) has helped to establish empirically the linkages between exposure to poor solid waste management and perceptions of associated health risks and self‐reported health conditions and health care sought in the 12 months preceding the studies (APHRC, 2017a, 2017b). Biomedical sampling around the municipal waste dump site in Nairobi assessed the prevalence of infections (hepatitis B, skin infections, intestinal infestations), injuries and accidents, as well as chronic and psychological illnesses associated with exposure to solid waste among waste workers at the Dandora dump site. Related to this, water sampling kits that can be used to record total dissolved solids, total coliform and faecal coliform were used to supplement the household survey and participatory tools in Karonga (Manda & Wanda, 2017), and found that more than half of the samples did not meet the World Health Organization's standards of being fit for domestic purposes prior to treatment – yet many households rely on these sources for drinking water.

Remote‐sensing techniques can also be used to build an understanding of the spectrum of urban risk. One approach to understanding urban risk in the light of the relative data paucity for many areas of Africa is by developing “good enough” models to create first approximations. For several Urban ARK cities, remote sensing was used to approximate the infrastructure typologies in different parts of the city in terms of which physical infrastructures are likely to be present (e.g., roads, water, communications, electricity) and what the level of service might be (e.g., unpaved roads, piped water, mobile telephones, electricity generators) (Bechtel et al., 2015; Taylor et al., 2017). By coarsely zoning the city into different parcels (“urban textures”), and in combination with coarse‐scale hazard maps, it is possible to estimate how different parts of a city might be affected differently by the same hazard event (Taylor et al., 2018). This may fall under the category of “models to learn with” or “models to play with” rather than giving precise, real‐time outputs – and indeed, this must be communicated carefully. But in cities where growth outstrips the pace of data collection, this coarse scale, first approximation helps to think of the city as a system of interconnected parcels, rather than one point, or a series of different case studies of different scales and approaches.

Globally available, medium–coarse‐scale datasets and frameworks can supplement local knowledge to build a deeper understanding of potential risks. A global framework of 21 natural hazards and their potential interactions (Gill & Malamud, 2014) has been used to systematically qualitatively assess the potential for each of those hazards to occur in the locality of Karonga based on a review of peer‐review and grey literature, supplemented with global datasets. This has shown that although certain commonly occurring hazards such as flooding are perceived as significant by urban residents, there is potential for other lower probability hazard impacts (e.g., extreme temperatures) and hazard interactions (e.g., earthquake‐triggered landslides). This approach has also been used in Nairobi to help inform planning for upgrading in informal settlements in the city. Datasets were shared with local policy makers, and were instrumental in the creation of the Nairobi Risk Partnership – a new effort to bring together evidence and policy for reducing risk in the city which is intended to ensure that the lessons from the programme continue to be applied after it formally ends. This process was led by the Nairobi City County with initial input from Urban ARK and involves local practitioners and other urban actors taking ownership and continuing to update and engage with such datasets and frameworks beyond research‐programme lifecycles.

## 7 CONCLUSION: PRINCIPLES FOR UNDERSTANDING RISK IN LOW‐INCOME URBAN CENTRES

**Figure 1:**
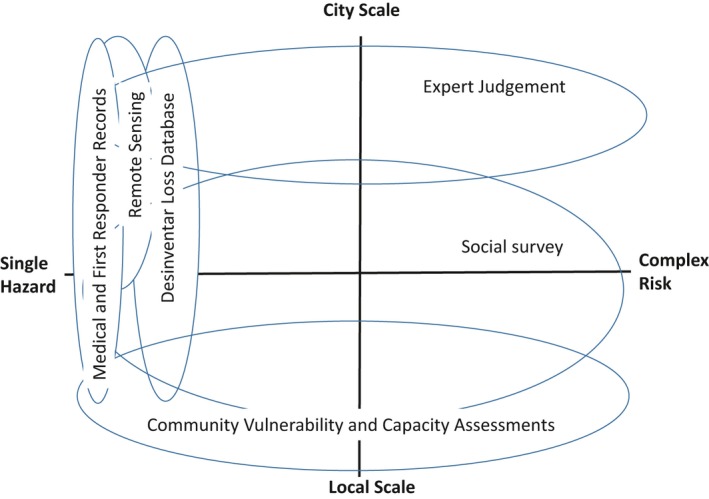
The spectrum of methods. [Colour figure can be viewed at wileyonlinelibrary.com]

Addressing the spectrum of urban risk requires innovation in methodological approaches: a diverse set of methods (quantitative and qualitative, deductive and inductive) and methodological pluralism capable of capturing and supporting action on risk in integrated ways. Adopting a multi‐ or mixed methods approach through a large programme such as Urban ARK helps to bridge technical, historical, social, health and other perspectives and data. The methodological approaches described in this paper each address an important, but partial, range of the spectrum of risk in urban centres in sub‐Saharan Africa. Rather than over‐emphasising their individual importance, therefore, it is important that they are valued and used in relation to this broad spectrum of risk. Taken together, they have the potential to address some of the major risks associated with urban growth in this setting. As illustrated in Figure 1, this approach goes beyond simply using multiple methods, but rather focuses on their integration to support a fuller understanding of risk and a more comprehensive and effective set of responses. It was supported by multi‐disciplinary teams working together in particular locations, by the coordinated sharing of information and approaches across a range of distinct but related contexts, and by an approach to research that was co‐developed with policy makers and other end users.

Several broad conclusions about how this might be achieved can therefore be drawn. First, methods are particularly useful where they are relevant locally. The approaches described above recognise the specific challenges posed by urbanisation in Africa and how these contribute to risk (Dodman et al., 2017), and identify strategies that can be used to understand these in low‐income neighbourhoods and high‐risk settlements. They also recognise the underlying political economy in urban centres and how this shapes the availability and reliability of data.

Second, doing this effectively requires integrative and inclusive approaches based on close partnerships between researchers, local community organisations, municipal authorities and other research users – not just to provide the type of information assumed to be useful, but to work closely in identifying data that will be useful for policy and practice. The methods described above have all sought to build on and enhance research capacity that exists within local research institutions, rather than eroding it through the provision of external “expertise,” at the same time as strengthening the ability of early career researchers to work collaboratively across a range of contexts.

Third, a pragmatic approach is often necessary. This can involve modifying methods from other contexts to suit the particular situation of low‐income urban centres, and recognising that deficiencies in data may require an innovative approach to data collection and analysis. This can also involve combining different data sources – including hospital records that provide quantitative information on the burden of disease and qualitative methods that provide information on underlying risk factors at the community level – to provide a more detailed and comprehensive picture of urban risk at different scales.

Finally, as well as having local applicability, there is a need for greater inter‐urban perspectives that identify and address risk differentials between urban centres of different sizes and in different geographic situations, and that can inform urban and regional approaches to planning and risk reduction. The mechanisms for sharing approaches and techniques between researchers in different African cities are being developed, but will require further support.

When these principles are put into practice, they can have significant and far‐reaching consequences. They can enhance technical responses to risk reduction through providing a stronger evidence base on the full spectrum of risks, making it less likely that policy makers will overlook or ignore less visible (but significant) threats. At the same time, by highlighting the value of participatory inputs to this process, a spectrum of risk approach can contribute to more transformative gains around improved urban governance and inclusion – responding to the vision of “no one left behind” from the Sustainable Development and “cities for all” from the New Urban Agenda.

## ENDNOTES

1 Urban ARK is a three‐year programme (2015–2018) funded by the Department for International Development (DFID) and Economic and Social Sciences Research Council (ESRC) – see www.urbanark.org.

2 The work reported in the subsequent sections has all been undertaken as part of, or associated with, the Urban ARK project described in the Introduction. Much of the information has been gathered directly by the authors of this paper. However, references are included to enable the reader to access further information on any of the methods or findings.
